# An Old Cholesterol Drug Could Help Clear PFAS

**DOI:** 10.1021/acscentsci.5c02346

**Published:** 2026-01-05

**Authors:** Saima Sidik

## Abstract

Highly exposed people have begun taking cholestyramine, but experts caution that evidence is lacking on whether the treatment improves long-term health.

## Graphical abstract

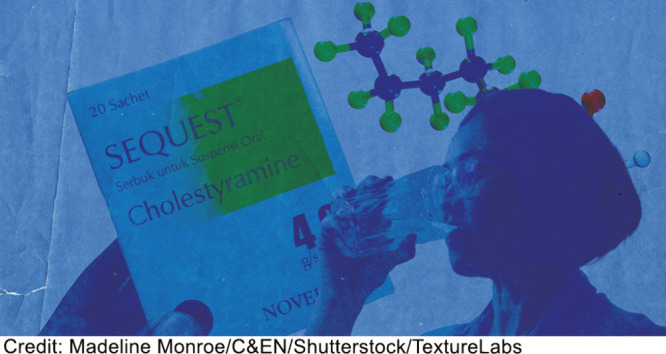

Paul Smith, a retired nurse anesthetist, has been contending with chronic lymphocytic leukemia since 2012, and his doctor suspects a tumor is forming in his pituitary gland. But despite the uncertainty about his health, a well-known cholesterol management drug is giving him a bit of hope.

Smith lives in a part of Barnstable, Massachusetts, called Centerville, where a local firefighter-training school and nearby airport contaminated the water supply for decades. The facilities allowed environmental leaks of fire-suppressing foam laden with per- and polyfluoroalkyl substances, also known as PFAS or “forever chemicals.”

Smith had been drinking the tainted water for years before he learned that it was sending his levels of certain PFAS soaring. In 2022, the Silent Spring Institute, a nonprofit health research organization, commissioned a study that included Centerville.

**Figure:**
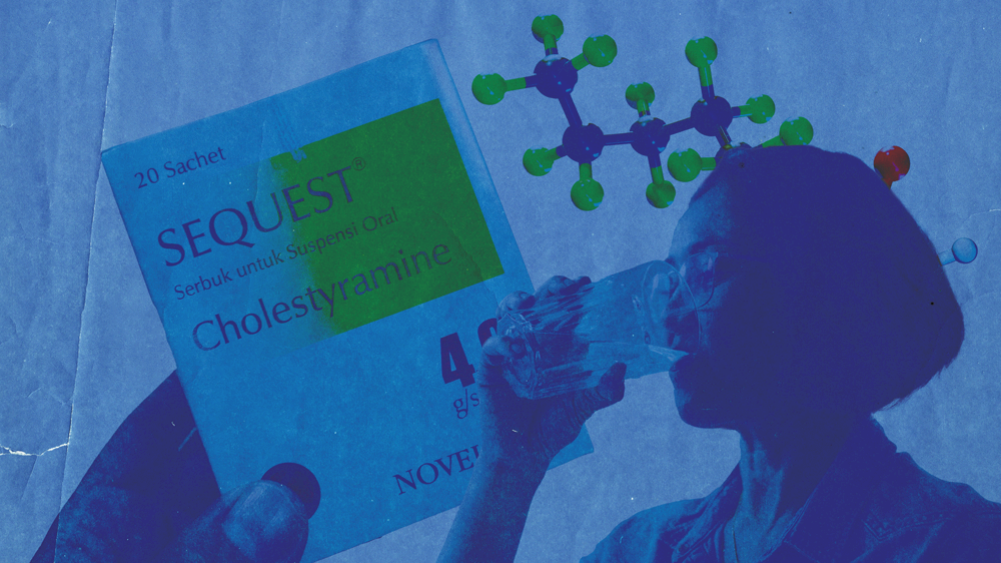
Credit: Madeline Monroe/C&EN/Shutterstock.

Through that study, Smith learned exactly how much of the PFAS were circulating in his blood. In short, his levels were high—in the case of one chemical, perfluorohexanesulfonic acid (PFHxS), his blood level was 14 times the national average (PDF). After he got the results, “I was feeling pretty bad,” he says.

And he’s not alone. Following a hefty exposure, PFAS levels can take decades to return to baseline; people like Smith often feel like they’re stuck carrying these toxic substances, which have been linked to health problems ranging from high cholesterol to cancer. Many people “want some sort of medical solution,” says epidemiologist Shiwen Li from the University of Hawaii at Manoa, who studies the health impacts of PFAS.

**Figure:**
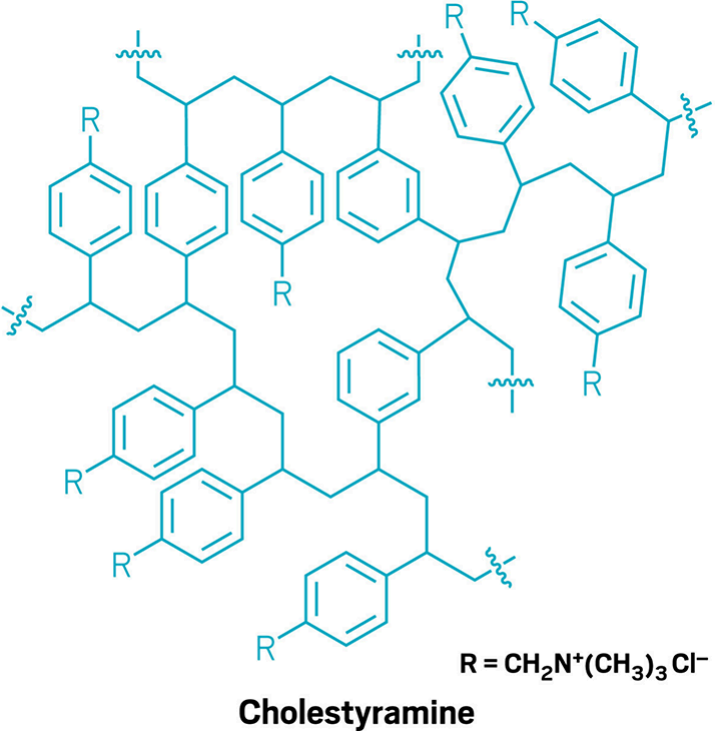
Cholestyramine is a tangled ion-exchange resin. When taken orally, it lingers in the gut and swaps its chloride ions for negatively charged bile acids, binding them.

Cholestyramine, a cholesterol management drug that was approved in the US more than 50 years ago, may be just that. Over the past 4 years, three studies have suggested that cholestyramine can eject PFAS from the body.

All three studies are preliminary, cautions Li, who was not involved any of them. In his opinion, celebrating is premature. “Without larger clinical trials, we shouldn’t be advertising that this is the solution.” At the same time, based on his knowledge of biochemistry, he says the mechanism of how the drug would remove PFAS makes sense.

Smith sought out cholestyramine after finding publications in the scientific literature about its potential benefit for people with high PFAS exposure. Smith’s gastroenterologist was familiar with cholestyramine and considered it safe, so he agreed to give Smith a prescription.

After about 8 months of taking cholestyramine, Smith has seen his PFAS levels drop dramatically. Two fluorinated chemicals became almost undetectable in his blood, and PFHxS dropped to about 30% of what it once was. “It was definitely a psychological boost,” he says.

Cholestyramine is a powdered resin taken orally that sticks around in the gut for a while, and it is thought to lower PFAS levels the same way that it lowers cholesterol: by interrupting bile acids’ round-trip voyage from the liver to the gut and back.

Normally, bile acids are made in the liver and then migrate to the gut, where they bind to fats and help the body digest them. Some fats can slow the body’s breakdown of cholesterol, so lowering levels of those fats can lower cholesterol. Cholestyramine accomplishes this by binding complexes of bile acids and fats while they’re in the gut, creating an insoluble matrix that leaves the body in feces. In the absence of cholestyramine, bile acids head back to the liver after they’re done in the gut so that the body can reuse them.

But PFAS can get swept up into this cycle too. Some PFAS share structural features with fats—namely, they both have long, neutrally charged tails and negatively charged heads—so PFAS can also bind to bile acids and ride along as they cycle between the liver and the gut. By breaking that cycle, cholestyramine can capture PFAS and expel them from the body into the toilet.

Since at least the 1980s, researchers have suspected that cholestyramine could lower PFAS levels, but scientists have only recently begun to test the idea rigorously. In a 2021 publication, Alan Ducatman, a long-time PFAS researcher at West Virginia University, followed up on the cholestyramine theory by reanalyzing data from a seminal study that linked PFAS levels to numerous health problems. He found that 36 of the study’s 56,175 participants happened to be taking cholestyramine, and their levels of certain PFAS were reduced compared with levels in the rest of the population. One PFAS in particular, perfluorooctanesulfonic acid (PFOS), “just really went away,” Ducatman says.
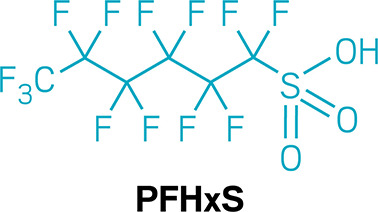
 
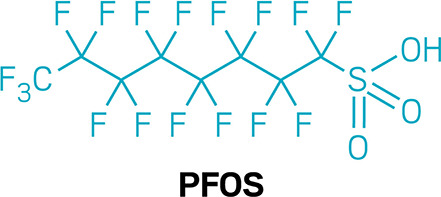


Meanwhile, in Denmark, nephrologist and internist Morten Lindhardt from the University of Copenhagen and his colleagues tested cholestyramine in a group of 45 people who were highly exposed to PFAS. The forever chemicals got in their systems after they ate meat from cattle that were raised near a firefighting school. Half the participants took cholestyramine for 12 weeks while the other half went untreated, and then the two groups switched.

Levels of various PFAS dropped by about 20–60% when study participants took cholestyramine. Levels in the untreated group dropped only about 3%, or, in some cases, rose slightly. “The effect of treatment was astronomical—huge effect,” Lindhardt says.

Around the same time, firefighters in Australia were starting to realize the extent to which firefighting foam might endanger their health. In response, in early 2018, the South Australian Metropolitan Fire Service launched a voluntary program to identify firefighters who had been highly exposed and offer treatment to them. Twelve firefighters chose to take cholestyramine, and their levels of PFOS and PFHxS seemed to drop about three times as much as levels in those who went without treatment over the course of a year.

It’s important to note, however, that the study in Australia was designed around the desires of affected people rather than around producing robust scientific findings, says toxicologist Ian Delaere, an author on the study and the director of the toxicology consultancy Expotox. Because of this design decision, the study is small and comes with many caveats.

For example, participants may have begun avoiding PFAS in their daily lives because they knew their levels were high, making cholestyramine look more effective than it actually is, Li says. But when you look at the results next to other recent studies, “they all seem to be heading in the same direction,” Delaere says.

Cholestyramine often causes gastrointestinal side effects such as nausea and diarrhea, and might cause the body to excrete fat-soluble vitamins. “It’s not wonderfully pleasant, but it’s not particularly dangerous,” Ducatman says. Lindhardt thinks tinkering with the dose of cholestyramine and length of treatment could reduce the chance of side effects and limit how long users have to tolerate them.

But other experts warn that cholestyramine’s initial effectiveness at lowering blood PFAS levels could be deceptive. Just because levels of PFAS drop in a person’s blood does not necessarily mean they drop in all tissues, Li says. Over time, PFAS may leach out of tissues such as fat and into the blood, causing levels to go up again.

Whether reducing PFAS levels after a hefty exposure has long-term health benefits is a topic of discussion within the research community. It is possible that once the chemicals have entered a person’s body, the damage has been done, and clearing them has little impact.

Lindhardt thinks that anyone who argues for the health benefits of using cholestyramine to speed up PFAS clearance is “on thin ice” at this early stage in the research. He compares the situation to smoking: “Your risk of having lung cancer is not eliminated the day after you stop,” he says. But by the same logic, Delaere counters, telling a person not to use cholestyramine to reduce their PFAS levels would be like telling a smoker that there’s no health benefit to quitting, which is clearly untrue.

There is some indication that “your body can slowly cure itself” after PFAS are cleared, Li says. So far, the evidence centers around cholesterol levels, which go up in response to PFAS exposure. As PFAS levels drop, cholesterol levels also appear to return to normal.

Smith is hoping the same will hold true for his health problems. “If my levels come down, maybe some of these other issues I have will get better,” he says. And even if cholestyramine does not live up to its promise, he and his friends are already contending with serious health problems, so “what do we have to lose?” he asks.

*Saima Sidik is a freelance contributor to* Chemical & Engineering News, *the independent news outlet of the American Chemical Society.*

